# Immune memory reactivation and T cell dynamics following 12-month homologous CoronaVac booster: a longitudinal cohort study

**DOI:** 10.3389/fimmu.2025.1636629

**Published:** 2025-07-17

**Authors:** Xinran Song, Weixin Chen, Shuang Bai, Min Lv, Jian Wang, Ao Zhang, Jiang Wu, Wei Zhao

**Affiliations:** ^1^ Beijing Key Laboratory of Surveillance, Early Warning and Pathogen Research on Emerging Infectious Diseases, Beijing Center for Disease Prevention and Control, Beijing, China; ^2^ Beijing Research Center for Respiratory Infectious Diseases, Beijing, China; ^3^ School of Public Health, Capital Medical University, Beijing, China

**Keywords:** SARS-CoV-2 inactivated vaccine, homologous boosting, neutralizing antibodies, T cell exhaustion, Th1 polarization

## Abstract

**Background:**

Inactivated COVID-19 vaccines exhibit more rapid declines in antibody levels than other vaccine platforms, likely owing to transient antigen exposure and limited germinal center persistence. Moreover, although homologous boosting effectively restores humoral immunity, concerns persist regarding potential T cell exhaustion with repeated antigen exposure. We evaluated the effectiveness of delayed homologous CoronaVac booster immunization in reactivating immune memory.

**Methods:**

A prospective longitudinal cohort study was conducted with 83 healthy adults who received two CoronaVac vaccine doses (14-day interval) and a homologous booster shot after 12 months. Peripheral blood samples were collected 0, 3, 7, 10, and 14 days after booster vaccination. Neutralizing antibodies were analysed using live-virus microneutralization assays. Anti-receptor-binding domain immunoglobulin subclasses (IgG1, IgG2, IgG3, IgG4) were detected using enzyme-linked immunosorbent assay. Cytokine secretion (interferon [IFN]-γ/interleukin [IL]-2/IL-4/IL-5) was assessed using enzyme-linked immunospot assay. T cell polarization and exhaustion markers (T-bet/GATA3 and CD69/CTLA-4/PD-1) were evaluated using flow cytometry.

**Results:**

The geometric mean titer of neutralizing antibodies reached 254.5 on day 14. The initial immune response was dominated by IgG3, which subsequently shifted to IgG1. A significant Th1-type cellular immune response was characterized by increased IFN-γ and IL-2 secretion, and upregulated T-bet expression. Transient CD69+ T cell activation occurred between days 3 and 10 without sustained PD-1 and CTLA-4 elevation.

**Conclusions:**

Delayed homologous CoronaVac booster immunization effectively reactivates immune memory, facilitated by Th1 polarization and transient T cell activation, which do not result in T cell exhaustion. These findings suggest the potential application of long-interval immunization strategies against COVID-19.

## 1 Introduction

Global vaccination initiatives against SARS-CoV-2 have significantly reduced the incidence of severe COVID-19 and associated mortality (1, 2) However, persistent vaccine-induced immunity remains a critical challenge (3, 4). Across all vaccine platforms, neutralizing antibody titers decline over time, necessitating a comprehensive understanding of the mechanisms underlying immunological memory. mRNA-based vaccines (e.g., BNT162b2) confer prolonged protection through robust germinal center reactions and high-affinity memory B cell generation facilitated by intracellular antigen expression and dual major histocompatibility complex (MHC)-I/II pathway activation (5). Inactivated COVID-19 vaccines (e.g., CoronaVac) primarily elicit Th1-biased immune responses via pattern recognition receptor activation, characterized by transient antigen exposure and prominent interferon (IFN)-γ and interleukin (IL)-2 secretion profiles (6–8). Wang et al. (9). examined the safety and immunogenicity of a third CoronaVac dose in children and adolescents, revealing that a booster administered 10–12 months after the second dose significantly elevated neutralizing antibody levels against both wild-type SARS-CoV-2 and Omicron strains.

Inactivated COVID-19 vaccines incorporate multiple conserved SARS-CoV-2 antigens, such as the S, N, and E proteins, thereby inducing broad T cell responses against less mutable viral epitopes. This multi-antigen approach enhances cross-reactivity against emerging variants of concern, rendering inactivated vaccines less susceptible to antibody escape mutations. Inactivated vaccines continue to be widely available in China and other countries, despite exhibiting more rapid declines in antibody levels than do other vaccine platforms, likely due to transient antigen exposure and limited germinal center persistence (10). Moreover, although homologous boosting effectively restores humoral immunity, concerns persist regarding potential T cell exhaustion with repeated antigen exposure, a phenomenon documented in frequent influenza vaccinations, where excessive boosting may paradoxically compromise protective immunity (11, 12). In SARS-CoV-2 infection, elevated expression of exhaustion markers (PD-1 and CTLA-4) in CD8+ T cells correlates with disease severity (13, 14). Recent evidence suggests that repeated mRNA vaccination does not induce T cell dysfunction, as SARS-CoV-2-specific CD8+ T cells retain their proliferative capacity despite the transient expression of exhaustion markers (15). However, whether inactivated COVID-19 vaccines, which predominantly drive Th1-biased responses, similarly avoid long-term T cell exhaustion, remains unclear (16). This gap in knowledge is critical, as prolonged antigen-free intervals under extended-interval boosting (e.g., 12 months) may influence memory T cell differentiation and exhaustion.

Previous studies have predominantly focused on the short-term effects of boosters (within 6 months), leaving three unresolved questions central to extended-interval strategies: (1) whether prolonged intervals permit memory T cell differentiation into less exhaustible subsets, preserving functionality; (2) how delayed boosting shapes IgG subclass dynamics and their correlation with neutralization potency; (3) whether extended intervals maintain the Th1/Th2 equilibrium that is essential for antiviral efficacy while avoiding immunopathology. Additionally, temporal coordination of early T cell activation (e.g., CD69 upregulation) and late antibody maturation in long-interval boosting remain unexplored, despite their implications for immune safety and memory reactivation efficiency.

To address these issues, this study aimed to delineate (1) the reactivation kinetics of humoral and cellular memory, (2) Th1/Th2 polarization dynamics, and (3) the risk of T cell exhaustion under extended-interval boosting in adults who received a homologous CoronaVac booster vaccine 12 months after primary immunization. Our findings provide critical insights into the optimization of inactivated vaccine strategies for durable protection, particularly in resource-limited settings where extended-interval boosting may be necessary.

## 2 Materials and methods

### 2.1 Study design and ethical considerations

**Figure 1:**
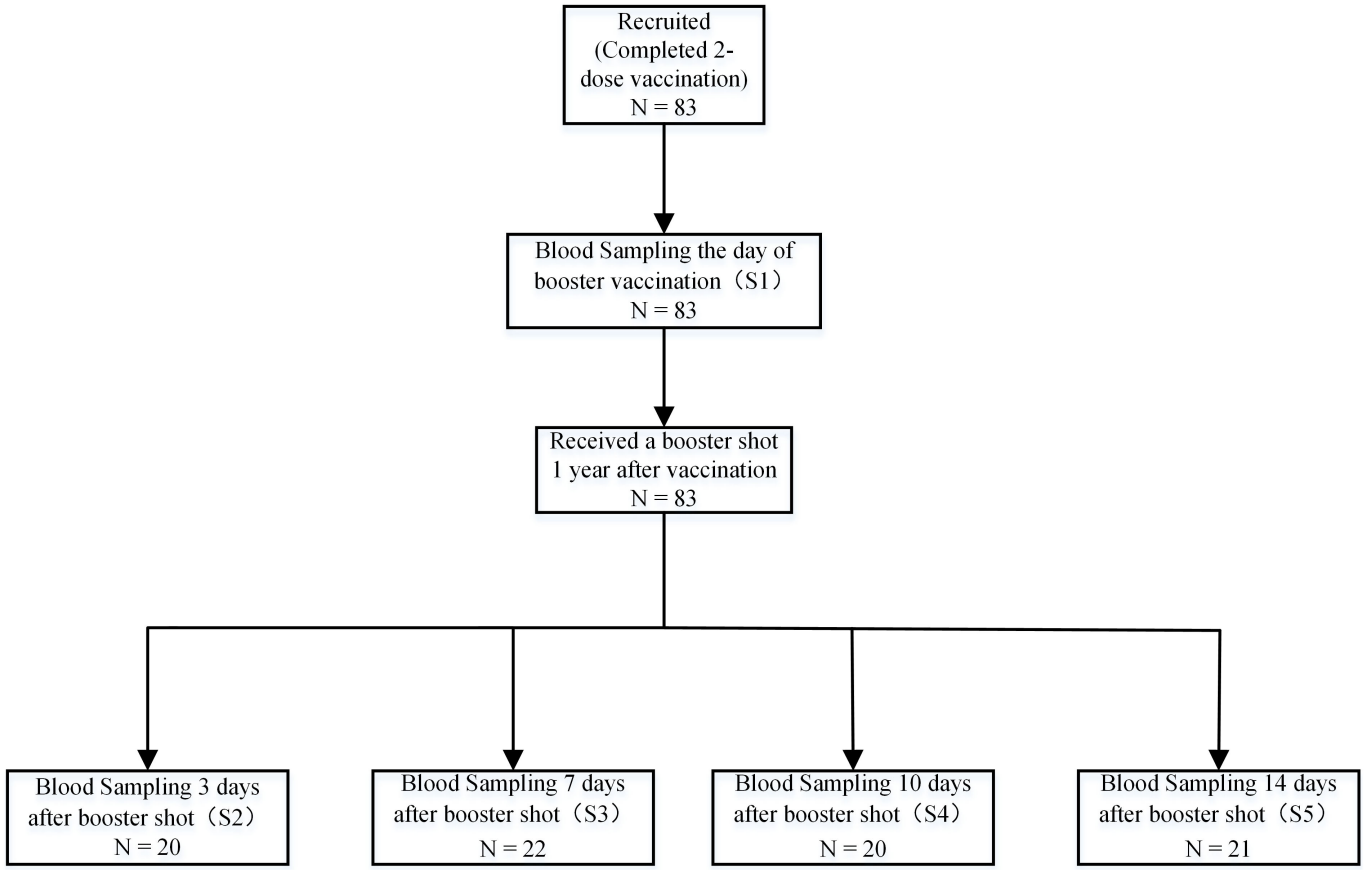
Sample collection schedule. A total of 83 participants aged 22 to 57 were enrolled at the Beijing CDC in China. Participants received a two-dose vaccination regimen of 3 mg CoronaVac, administered intramuscularly with a 14-day interval prior to enrollment. This was followed by a homologous booster immunization administered 12 months after the second dose. Samples, including serum, plasma, and peripheral blood mononuclear cells, were collected at day 0 (baseline) before the booster and on days 3, 7, 10, and 14 post-booster. A total of 83 participants completed the endpoint study.

This prospective cohort study, conducted from May 10, 2021, to May 10, 2022, evaluated the immunogenicity of CoronaVac (Sinovac) in healthy adults aged 22 to 57 years in Beijing. Eligible non-pregnant participants provided informed consent prior to enrollment. The study protocol was approved by the Beijing CDC Ethics Committee (Approval No: 2020-28) and adhered to the principles of Chinese Good Clinical Practice and ICH guidelines. Exclusion criteria included a history of prior SARS-CoV, SARS-CoV-2, or MERS infection, high-risk epidemiological exposure within 14 days of vaccination, axillary temperature exceeding 37.0°C, or known allergy to any vaccine component; all participants were screened for chronic conditions (e.g., diabetes, immunosuppression, renal failure) and excluded if those conditions were clinically significant. Participants received two doses of the vaccine on days 0 and 14, followed by a homologous booster at 12 months. Following the booster, subjects were randomized into four groups, with serum samples collected at baseline (day 0), and at days 3, 7, 10, and 14. Both peripheral blood mononuclear cells and serum were analyzed to assess exploratory endpoints (**Figure 1**).

### 2.2 PBMC and serum collection

PBMC and serum collection procedures followed established protocols (17). Blood samples were collected in conventional/heparin-treated tubes and processed within 4 h. Serum was separated by centrifugation (1,800 rpm, 5 min) and stored at −80°C. PBMCs were isolated using Leucosep tubes (Greiner Bio-One, Germany) via Ficoll-Paque gradient separation, cryopreserved at −80°C, and stored in liquid nitrogen. Prior to analysis, PBMCs were thawed at 37°C and washed twice.

### 2.3 Anti-SARS-CoV-2 receptor-binding domain (RBD) IgG assay

Commercial chemiluminescence detection kits (2019-nCoV IgG antibody detection kit; Bioscience Diagnostics, Tianjin, China) were employed to measure SARS-CoV-2 RBD-specific IgG following the manufacturer’s instructions. The positive cutoff value for RBD-specific IgG antibodies was defined as a sample-to-cutoff ratio (S/CO) ≥ 1.0

### 2.4 Neutralization assay

The biological potency of SARS-CoV-2 neutralizing antibodies was quantified using a micro-cytopathic effect (CPE) assay with the wild-type strain (SARS-CoV-2/human/CHN/CN1/2020), as previously described (18). Briefly, heat-inactivated serum (56°C, 30 min) underwent serial dilution in DMEM containing 2% FBS. Diluted sera were incubated with 100 TCID50 viral inoculum (50 μL) at 37°C for 2 h. Antigen-antibody complexes were then added to Vero E6 monolayers (1.5 × 10^5^ cells/mL) and cultured at 36.5°C/5% CO_2_ for 120 h. CPE was assessed microscopically, with 50% neutralization titers (NT50) calculated via the Reed–Muench method. Serum seroconversion was defined as NT50 ≥ 8 (19), indicating > 50% cellular protection at 1:8 dilution.

### 2.5 Anti-SARS-CoV-2 receptor-binding domain (RBD) IgG subclasses assay

SARS-CoV-2 RBD-specific IgG subclass levels (IgG1, IgG2, IgG3, IgG4) were quantified using an ELISA kit (ACROBiosystems). Samples were initially diluted 1:20 to balance sensitivity and background reduction. The cutoff (0.1) was defined as 2.1× the mean OD (Optical Density) of SARS-CoV-2-negative controls. Positivity was determined by S/CO ratio (sample OD/cutoff ≥ 1). Antibody titers were calculated as S/CO × dilution factor. Samples exceeding the standard curve’s linear range underwent serial dilution until negativity, with the highest valid dilution used for quantification.

### 2.6 Ex vivo ELISpot assay

Cryopreserved PBMCs were stimulated with SARS-CoV-2 spike-derived synthetic non-structural multimeric oligomer (SNMO, 2 μg/ml; Mabtech AB #3622-1), a 47-peptide HLA-binding pool spanning S/N/M/ORF3a/ORF7a proteins. IFN-γ/IL-2/IL-4/IL-5 ELISpot assays were performed using 2 × 10^5^ PBMCs per well (16–18 h incubation). Antigen-specific responses were calculated by subtracting control well spot counts from stimulated wells, expressed as spot-forming units (s.f.u./10^6^ PBMCs). Positive responses required: 1) ≥ 3× mean negative control values, 2) > 25 s.f.u./10^6^ PBMCs, and 3) confirmation through replicate testing. Data exclusion criteria: negative controls > 30 s.f.u./10^6^ PBMCs or failed phytohemagglutinin-positive controls.

### 2.7 Flow cytometry experiment

For multiparametric flow cytometry analysis, each sample was divided into three parallel staining panels to prevent spectral overlap and optimize antibody combinations. Panel 1 focused on intracellular transcription factors. Peripheral blood mononuclear cells (PBMCs) were stimulated with SARS-CoV-2 SNMO (catalog #3622-1; Mabtech AB, Stockholm, Sweden) for 20 h under 5% CO_2_ at 37°C to activate antigen-specific T cells. After viability staining using Fixable Viability Stain 780 (BD Horizon™), the cells were fixed with pre-warmed BD Cytofix™ (37°C, 10 min), permeabilized with ice-cold BD Phosflow™ Perm Buffer III (30 min on ice), and stained with antibodies against BUV510-anti-CD3, PE-anti-CD4, FITC-anti-CD8, BV421-anti-T-bet, and Alexa Fluor^®^ 647-anti-GATA3 (all from BD Biosciences, Franklin Lakes, NJ, USA) in Brilliant Stain Buffer (Cat. 566349). Panel 2 assessed surface activation markers. After viability staining with Fixable Viability Stain 780 (BD Horizon™), cells were labeled with CD3, CD4, CD8, and BV421-anti-CD69. Panel 3 focused on exhaustion markers. Cells were stained with CD3, CD4, CD8, APC-anti-PD-1, and BV421-anti-CTLA-4. All panels included a 30-min incubation with antibodies at 4°C, followed by two washes in BD Pharmingen™ Stain Buffer (Cat. 554656). Data acquisition was performed on a BD FACsymphony A5™ cytometer, and compensation was adjusted using single-stained controls. Analysis was conducted using the FlowJo™ v10.8 software.

### 2.8 Safety assessment

Participants were monitored for immediate adverse events (AEs) within 30 min following booster vaccination. All individuals (n = 83) were provided with standardized diary cards to document any local or systemic AEs occurring within 28 days post-vaccination. Solicited local AEs included injection site pain, induration, erythema, and pruritus, while solicited systemic AEs encompassed fever, fatigue, headache, myalgia, nausea, and cough. Participants were instructed to actively record solicited events daily during the initial 7 days. Investigators conducted structured telephone follow-ups on days 3, 7, 14, and 28 to verify diary entries and capture unsolicited AEs. All reported events were classified according to the National Medical Product Administration severity grading criteria (Grade 1: mild; Grade 2: moderate; Grade 3: severe). No additional safety assessments beyond routine monitoring were implemented, as this study was focused on primary efficacy endpoints.

### 2.9 Statistical analysis

The levels of IgG subclasses are presented as the logarithmic transformation of the sample to cut-off ratio (S/CO) multiplied by the dilution factor, along with their corresponding 95% confidence intervals (CIs). Neutralizing antibody titers are expressed in terms of dilution factors, The geometric mean titers (GMTs) and their corresponding 95% confidence intervals were derived by calculating the logarithmic values of the titers followed by an antilogarithmic transformation. This study employed one-way analysis of variance (ANOVA) for data analysis, with a logarithmic transformation applied as a preprocessing step. A two-sided p-value < 0.05 was considered statistically significant. All statistical analyses were conducted using the GraphPad Prism 8.0.1 software.

## 3 Results

### 3.1 Participants

The 83 participants (22–57 years, mean age 37.9 years) were randomly assigned to four study groups. Demographic analysis (**Table 1**) revealed homogeneity between the groups. The Kruskal–Wallis test showed no statistically significant differences in mean age among the groups (P = 0.156), ranging from 34.7 years (14-day post-vaccination group) to 41.2 years (3-day post-vaccination group). The sex distribution varied from 40.0 to 63.6% male participants across groups, with no statistically significant differences (χ² test, P = 0.422). All participants were of the Han ethnicity.

**Table 1:** Characteristics of the study participants.

Characteristics	3-days post-vaccination	7-days post-vaccination	10-days post-vaccination	14-days post-vaccination	P-value
	(N = 20)	(N = 22)	(N = 20)	(N = 21)	
Age (years)					0.156†
Mean (SD)	41.2 (7.75)	36.82 (8.89)	39.3 (9.20)	34.67 (6.37)	
Median	40	37	38	35	
Min, Max	28,53	22,57	27,56	26,47	
Sex, n (%)					0.422‡
Male	8(40)	14(63.6)	11(55)	10(47.6)	
Female	12(60)	8(36.4)	9(45)	11(52.4)	
Ethnicity [n (%)]					
Han	20(100)	22(100)	20(100)	21(100)	

SD, standard deviation.

†, Kruskal–Wallis test.

‡, Chi-square test.

### 3.2 Dynamics of humoral immune response following booster vaccination

**Figure 2:**
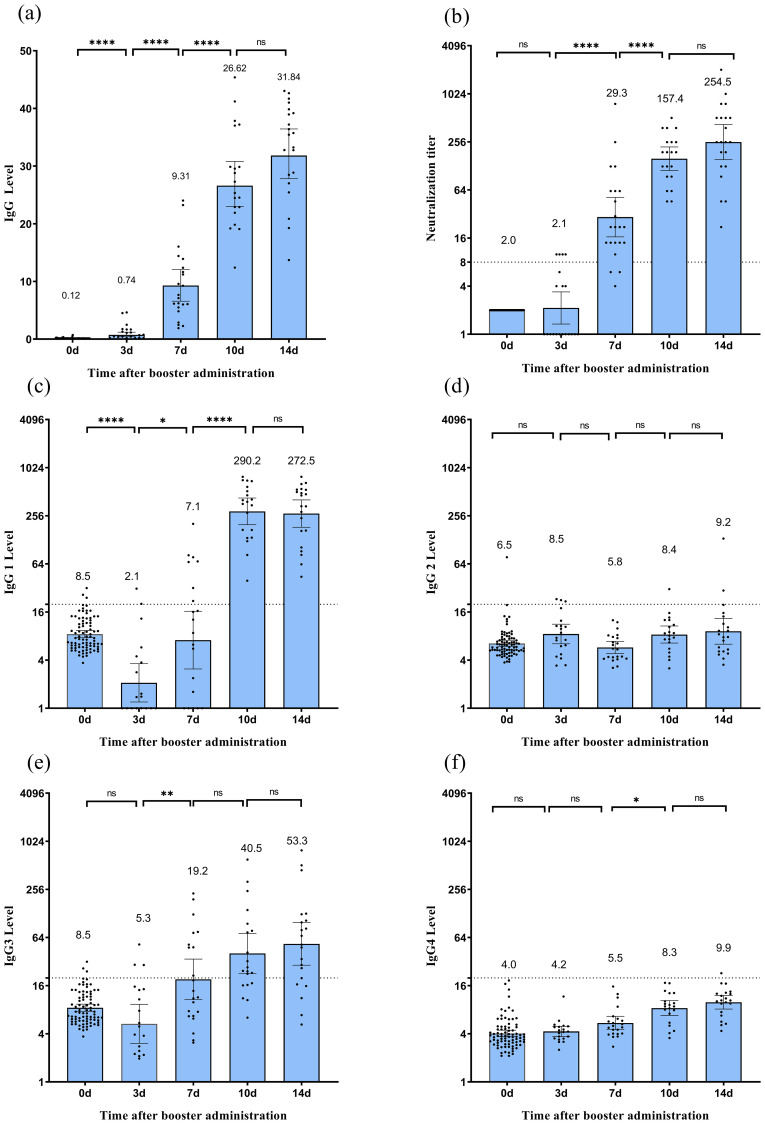
Humoral immune response following CoronaVac vaccination. **(a)** Spike RBD-binding IgG measured by CLIA.**(b)** Neutralizing antibody titers against the SARS-CoV-2 wild-type strain measured by microcytopathogenic effect assay. **(c–f)** Levels of IgG1 **(c)**, IgG2 **(d)**, IgG3 **(e)**, and IgG4 **(f)** subclasses measured by ELISA. Each dot represents an individual serum sample. For Spike RBD-binding IgG, the error bars of binding antibody are the mean with 95% CI. For neutralizing antibodies, error bars indicate geometric mean titers (GMT) with 95% confidence intervals (CI). Titers below the limit of detection (1:4) are plotted as half the detection limit (1:2). The dashed horizontal line denotes the seropositivity threshold (titer ≥1:8). For IgG subclasses, error bars represent arithmetic means with 95% CI. The dashed line denotes the seropositivity threshold of 20. Statistical significance between time points is indicated by asterisks (*p < 0.05, **p < 0.01, ****p < 0.0001; Wilcoxon matched-pair signed-rank test). CLIA, chemiluminescent immunoassay.

Booster vaccination rapidly activated immune memory and elicited a robust humoral response (**Figure 2b**). At baseline (day 0), the geometric mean titer (GMT) of neutralizing antibodies was 2.0 (95% CI: 2.0–2.0), with no seroconversion detected (0%). By day 3 post-vaccination, GMT increased to 2.1 (95% CI: 1.3–3.4), with 20.0% seroconversion. Subsequently, antibody levels exhibited exponential growth: by day 7, GMT significantly increased to 29.3 (95% CI: 16.6–51.8, P < 0.05) with 86.4% seroconversion, by 10 day had surged to 157.4 (95% CI: 112.1–221.1, P < 0.05) with 100% seroconversion, and by day 14 remained elevated at 254.5 (95% CI: 153.3–422.5), showing no statistically significant increase from day 10 (P > 0.05), with the seroconversion rate consistently maintained at 100%. Longitudinal analysis revealed concordant kinetic profiles between receptor-binding domain (RBD)-specific IgG antibody levels quantified by chemiluminescent immunoassay (CLIA) and neutralizing antibody titers over time. This dynamic pattern aligns with previous research (20), confirming that booster vaccination effectively reactivates memory B cells, counteracts natural declines in antibody titers following primary immunization, and facilitates the rapid reconstruction of immune responses.

ELISA analysis revealed that IgG1 and IgG3 were the predominant subclasses (**Figure 2**). As illustrated in **Figures 2c, e**, IgG1 levels decreased to 8.5 (95% CI: 7.7–9.4) at 12 months post-second vaccine dose, significantly dropped to 2.1 (95% CI: 1.2–3.6, P < 0.05) on day 3 post-booster, increased to 7.1 (95% CI: 3.1–16.4, P<0.05) by day 7, dramatically surged to 290.2 (95% CI: 197.6–426.2, P < 0.05) by day 10, and slightly decreased to 272.5 (95% CI: 183.7–404.0, P > 0.05) by day 14. In contrast, the average IgG3 level slightly decreased to 5.3 (95% CI: 3.0–9.3, P = 0.29) on day 3 post-booster; subsequently, it significantly increased to 19.2 (95% CI: 10.7–34.4, P < 0.05) by day 7, and increased again to 40.5 (95% CI: 22.8–71.8, P > 0.05) by day 10. By day 14 post-immunization, it reached 53.5 (95% CI: 28.8–98.5, P > 0.05). As shown in **Figures 2d, f**, although the average levels of IgG2 and IgG4 exhibited fluctuating trends, they demonstrated a limited induction of expression. IgG1/IgG4 values were 1.3 on day 7, 35.0 on day 10, and 27.5 on day 14.

### 3.3 Cellular immune polarization and cytokine coordination

**Figure 3:**
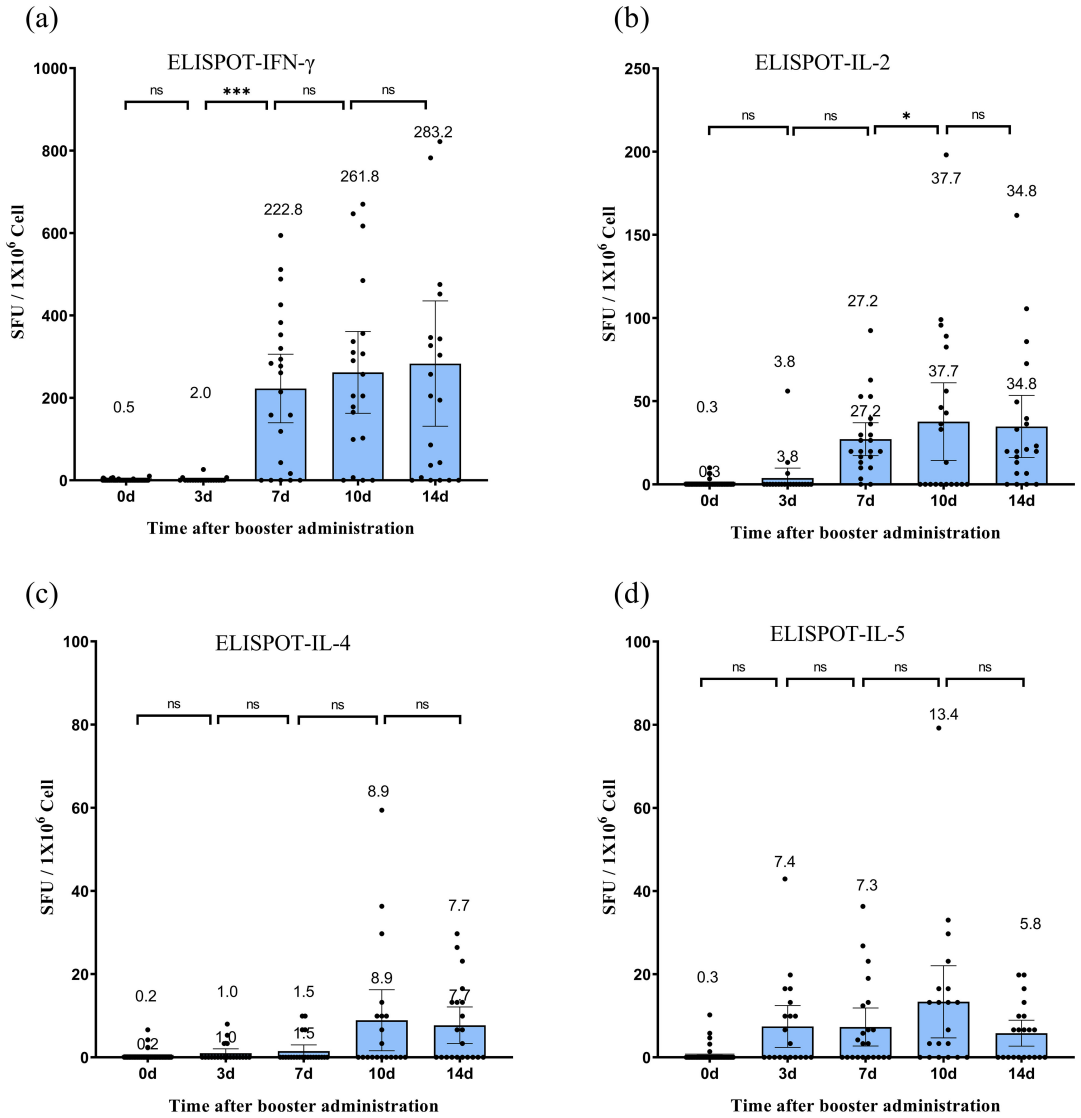
Specific T-cell responses following CoronaVac vaccination. **(a–d)** Frequencies of SARS-CoV-2-specific T cells secreting IFN-γ **(a)**, IL-2 **(b)**, IL-4 **(c)**, and IL-5 **(d)** quantified by *ex vivo* ELISpot assay. Peripheral blood mononuclear cells (PBMCs) were isolated from participants before vaccination (day 0) and on days 3, 7, 10, and 14 post-vaccination. Data are expressed as spot-forming units (SFU) per 10^6^ PBMCs after subtracting background values from unstimulated controls. Each data point represents the mean of triplicate wells for one participant. Horizontal lines above bars indicate geometric means, with error bars representing 95% confidence intervals (CI). Statistical significance between time points was determined by the Wilcoxon matched-pair signed-rank test (*p < 0.05, ***p < 0.001; two-sided *p*-values). IFN, interferon; IL, interleukin; SFU, spot-forming units.

IFN-γ secretion exhibited a continuous upward trend following the booster dose (**Figure 3**), increasing from nearly negligible levels at day 0 to 283.2 (95% CI: 131.1–435.2, P < 0.05) SFU/1 × 10^6^ cells by day 14. IL-2 secretion increased significantly, peaking at 37.7 (95% CI: 14.3–61.1, P < 0.05) SFU/1 × 10^6^ cells on day 10, before slightly decreasing to 34.8 (95% CI: 16.1–53.5, P > 0.05) SFU/1 × 10^6^ cells on day 14. IL-4 secretion remained relatively low and fluctuated minimally, peaking at 8.9 (95% CI: 1.6–16.3) SFU/1 × 10^6^ cells on day 10. IL-5 secretion initially increased, reaching 13.4 (95% CI: 4.7–22.0) SFU/1 × 10^6^ cells on day 10, before declining to 5.8 (95% CI: 2.7–8.9, P > 0.05) SFU/1 × 10^6^ cells on day 14. Although some participants exhibited IL-4 and IL-5 responses post-vaccination (**Figures 3c, d**), the lower responses of IL-4 and IL-5 compared with those of IFN-γ and IL-2 at most time points after vaccination (except for IL-5 on day 3) indicated a Th1-biased cellular immune response.

**Figure 4:**
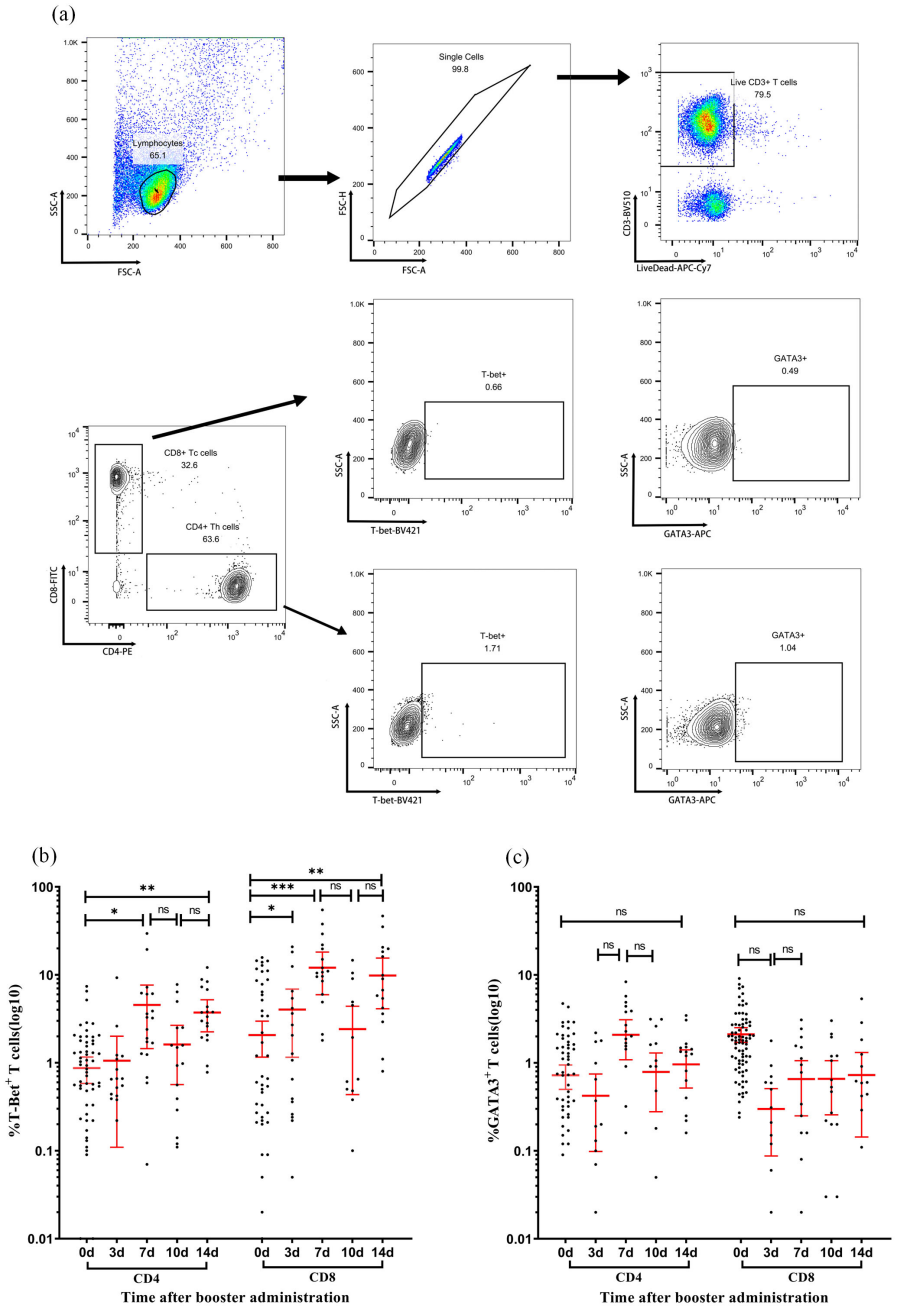
Dynamic expression of T-bet and GATA3 in T-cell subsets following immunization. **(a)** Representative flow cytometry gating strategy. Sequential identification of CD3+ T cells, CD4+ T cells, CD8+ T cells, and intracellular expression of transcription factors T-bet and GATA3 in CD4+ and CD8+ subsets. **(b, c)** Expression levels of T-bet **(b)** and GATA3 **(c)** in CD4+ T cells (left panels) and CD8+ T cells (right panels) measured at days 0, 7, 10, and 14 post-vaccination. Each data point represents an individual sample. Horizontal lines indicate arithmetic means, with error bars showing 95% confidence intervals (CI). Statistical significance between time points was determined by the Wilcoxon matched-pair signed-rank test (*p < 0.05, **p < 0.01, ***p < 0.001; two-sided *p*-values).

The expression of transcription factors T-bet and GATA3 in CD4 and CD8 T cells was examined at various time points post-immunization (**Figure 4**). In CD4+ T cells, the proportion of SARS-CoV-2 SNMO-specific T-bet increased to 4.55% (95% CI: 1.5–7.7) on day 7, which was significantly higher than that at baseline (P > 0.05). Subsequently, the proportion of T-bet decreased to 1.6% (95% CI: 0.6–2.7, P > 0.05) on day 10, before increasing to 3.7% (95% CI: 2.3–5.2, P > 0.05) on day 14. Similarly, CD8+ T cells exhibited a fluctuating T-bet expression pattern. On day 3 after booster vaccination, the proportion of T-bet increased to 4.0% (95% CI: 1.2–6.9), which was significantly higher than the baseline level (P = 0.01). On day 7, the proportion of T-bet increased again to 12.04% (95% CI: 5.9–18.2, P > 0.05), followed by a decrease to 2.4% (95% CI: 0.4–4.4, P > 0.05) on day 10, and then increased again to 9.8% (95% CI: 4.1–15.5, P > 0.05) on day 14. GATA3 levels remained low and stable in both CD4+ and CD8+ T cells, with no statistically significant differences between the time points. Collectively, the T-bet/GATA3 expression profiles suggested a dominant Th1-polarized response over Th2 activation, consistent with the IFN-γ-dominated cellular immunity observed in ELISpot assays.

### 3.4 Dynamic equilibrium of T cell activation and regulatory markers

**Figure 5:**
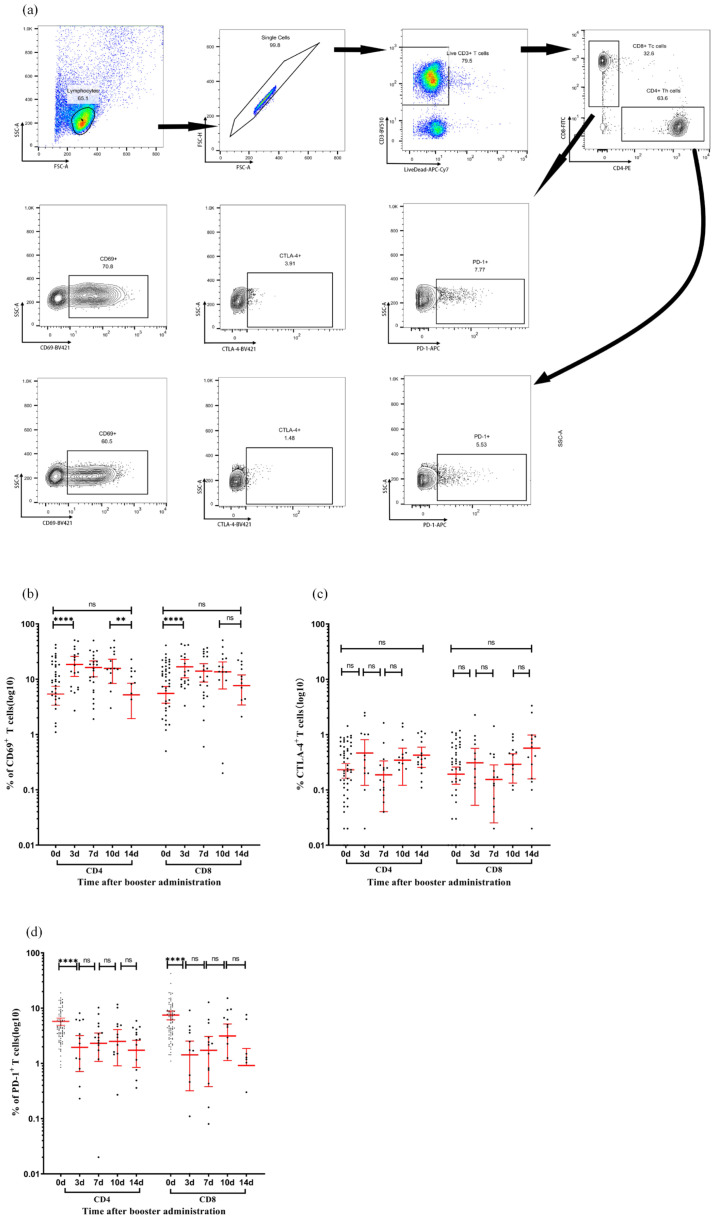
Activation and checkpoint marker dynamics in T-cell subsets following immunization. **(a)** Representative flow cytometry gating strategy. Sequential identification of CD3+ T cells, CD4+ T cells, CD8+ T cells, and intracellular expression of CD69, CTLA-4, and PD-1 in CD4+ and CD8+ subsets. **(b–d)** Expression percentages of CD69 **(b)**, CTLA-4 **(c)**, and PD-1 **(d)** in CD4+ T cells (left panels) and CD8+ T cells (right panels) measured at days 0, 7, 10, and 14 post-vaccination. Each data point represents an individual sample. Horizontal lines indicate arithmetic means, with error bars showing 95% confidence intervals (CI). Statistical significance between time points was determined by the Wilcoxon matched-pair signed-rank test (**p < 0.01, ****p < 0.0001; two-sided *p*-values). CI, confidence interval; CTLA-4, cytotoxic T-lymphocyte-associated protein 4; PD-1, programmed cell death protein 1.

Flow cytometry analysis revealed the temporal dynamics of activation and regulatory markers in the CD4+ and CD8+ T cell subsets after booster immunization (**Figure 5**). The proportion of CD69+ cells in CD4+ T cells significantly increased to 18.5% (95% CI: 11.2–25.8, P < 0.05) on day 3, maintained this level until day 10, and then declined to a baseline level of 5.2% (95% CI: 1.9–8.5, P < 0.05) on day 14. CD8+ T cells exhibited a similar trend, with the proportion of CD69+ T cells significantly increasing to 16.70% (95% CI: 10.6–22.8, P < 0.05) on day 3, maintained until day 10, and returned to baseline levels at 7.67% (95% CI: 3.4–11.9, P > 0.05) on day 14. CTLA-4 expression in both CD4+ and CD8+ T cells demonstrated a similar fluctuating pattern without statistically significant differences between time points. PD-1 expression in CD4+ T cells decreased to 1.95% (95% CI: 0.7–3.2, P < 0.05) on day 3, then fluctuated without significant differences through day 14, rising to 2.3% (95% CI: 1.1–3.5, P > 0.05) on day 7, 2.50% (95% CI: 0.9–4.1, P > 0.05) on day 10, and decreasing to 1.73% (95% CI: 0.9–2.6, P > 0.05) on day 14. In CD8+ T cells, PD-1 significantly decreased to 1.42% (95% CI: 0.3–2.5, P < 0.05) on day 3, and then varied without significant differences through day 14, rising to 1.72% (95% CI: 0.4–3.1, P > 0.05) on day 7, 3.14% (95% CI: 1.1–5.2, P > 0.05) on day 10, and declining to 0.91% (95% CI: −0.03 to 1.86, P > 0.05) on day 14. These findings suggest that the booster vaccine administered 12 months after the initial immunization effectively activated T cells without inducing an exhausted phenotype.

### 3.5 Safety

The booster vaccination demonstrated a favorable safety profile among all 83 participants. No immediate hypersensitivity reactions or vaccine-related severe adverse events (SAEs) were observed during the 30-min post-vaccination monitoring period. Throughout the 28-day follow-up, no participants reported solicited local or systemic AEs, nor were any unsolicited AEs or SAEs documented in diary cards or investigator interviews. All participants completed the full observation period without discontinuation owing to safety concerns. These findings align with prior studies indicating the well-tolerated nature of this vaccine platform in larger cohorts.

## 4 Discussion

Our findings demonstrate that delayed booster immunization effectively restores neutralizing antibody titers while inducing Th1-polarised cellular responses without triggering persistent T cell exhaustion. These observations offer critical insights into the immunological advantages of extended-interval inactivated vaccination.

The rapid resurgence of neutralizing antibodies following booster administration is consistent with previous reports of inactivated vaccine-induced memory B cell reactivation (20). Notably, the temporal dichotomy between the IgG3 and IgG1 subclasses reveals a sophisticated functional stratification. IgG3-dominated early responses (days 3–7) likely reflect extrafollicular plasmablast activation, utilizing its potent complement-binding capacity (C1q affinity >10-fold higher than that of IgG1) for immediate viral containment (21, 22). Conversely, IgG1 predominance from day 7 onwards correlated with germinal center-derived high-affinity antibodies, supported by sustained T follicular helper cell activity. The dynamic evolution of IgG subclass profiles following repeated vaccination revealed significant differences between the mRNA and inactivated vaccines. Irrgang et al (23).demonstrated that repeated mRNA vaccination, such as BNT162b2, induces a progressive class switch toward spike-specific IgG4 antibodies, with IgG4 proportions increasing from 0.04% post-second dose to 19.27% post-third dose. This phenomenon is attributed to prolonged germinal center activity, sustained antigen exposure, and sequential class-switch recombination toward distal IgG subclasses (γ4), facilitated by prolonged antigen expression and robust T follicular helper cell responses associated with mRNA vaccines. In contrast, inactivated vaccines, which deliver whole inactivated viral particles without persistent antigen production, predominantly elicit transient IgG1 and IgG3 responses with minimal IgG4 induction. This discrepancy may arise from differences in antigen presentation kinetics: mRNA vaccines promote prolonged B-cell receptor engagement and cytokine signaling (for example, IL-4/IL-10), driving IgG4 class switching, whereas inactivated vaccines likely favor short-lived plasmablast responses with limited germinal center maturation. Importantly, the low IgG4 levels observed with inactivated vaccines may preserve Fc-mediated effector functions (e.g., antibody-dependent cellular phagocytosis and antibody-dependent cellular cytotoxicity), which are critical for viral clearance but are diminished in IgG4-dominated responses (24). These findings underscore the need to evaluate the long-term functional implications of different vaccine platforms, particularly in the context of variant adaptation and booster strategies.

The robust Th1 bias observed in cellular responses, characterized by IFN-γ/IL-2 dominance and T-bet upregulation, with IgG1/IgG4 ratios > 1, is mechanistically associated with the aluminum hydroxide adjuvant-induced activation of NLRP3 inflammasome signaling in antigen-presenting cells (25). This pathway promotes IL-1β/IL-18 secretion, thereby creating a cytokine milieu that favors Th1 differentiation over Th2 responses. The oscillatory T-bet expression pattern in CD8+ T cells, peaking on days 7 and 14, suggests cyclical clonal expansion and contraction, a phenomenon previously associated with prolonged antigen presentation in lymph nodes. Notably, stable GATA3 levels across time points (< 5% variance) confirmed minimal Th2 skewing, mitigating theoretical concerns regarding vaccine-associated enhanced respiratory diseases.

The transient activation of CD69+ T cells, peaking at day 3, without sustained elevation of PD-1/CTLA-4, challenges the paradigm of “vaccine exhaustion” observed in frequent revaccination against influenza. CD69, an early activation marker rapidly induced upon antigen contact, reflects transient T cell engagement, rather than sustained stimulation. However, severe COVID-19 is characterized by pan-T cell hyperactivation and exhaustion. This discrepancy may arise from two key factors: 1) the 12-month interval allows memory T cell pools to regain functional plasticity, as evidenced by murine studies showing memory stem cell replenishment after antigen clearance (26); and 2) inactivated vaccine antigens lack replicative capacity, thereby limiting prolonged MHC-I presentation that drives terminal T cell exhaustion in natural infections. Although extending booster intervals may optimize the functional plasticity of the memory T-cell pool, it remains unclear whether shorter-interval vaccination necessarily induces exhaustion. to date, no study has directly demonstrated T-cell exhaustion following short-interval administration of CoronaVac inactivated vaccines. Nevertheless, based on the mechanisms of prolonged antigen exposure in exhaustion paradigms. it is reasonable to infer that short-interval repeated vaccination may poses a risk of T-cell exhaustion. These findings support extended boosting intervals as a strategy for preserving T cell functionality.

This study had some limitations. First, the analysis of immune memory reactivation post-12-month homologous CoronaVac booster was restricted to days 0, 3, 7, and 14, which may not fully capture dynamic immune response trajectories. Second, the absence of cross-protective efficacy assessments against variants and long-term immune memory durability tracking limits insights into the comprehensive effectiveness of the vaccine. Finally, the small cohort size and exclusive focus on the Han population may constrain the generalizability of inactivated vaccine-induced immune features across diverse demographics. These issues are currently being addressed through ongoing clinical trials.

In summary, this study demonstrates that a delayed homologous CoronaVac booster at 12-months effectively reignites neutralizing antibody maturation and Th1-polarised cellular immunity without inducing persistent T cell exhaustion, despite waning humoral responses after primary vaccination. Transient CD69+ T cell activation and stable PD-1/CTLA-4 profiles underscore the immunological safety of extended boosting schedules, offering a strategic framework to balance antiviral efficacy and long-term immune resilience in public health. Future research should focus on investigating cross-protective efficacy against variants, conducting long-term immune memory durability tracking, expanding to diverse demographics, and further exploring the mechanisms underlying observed immune responses.

## Data Availability

The original contributions presented in the study are included in the article/supplementary material. Further inquiries can be directed to the corresponding author.
